# Critical linkages between land use change and human health in the Amazon region: A scoping review

**DOI:** 10.1371/journal.pone.0196414

**Published:** 2018-06-12

**Authors:** Molly Mastel, Alejandra Bussalleu, Valerie A. Paz-Soldán, Gabriela Salmón-Mulanovich, Armando Valdés-Velásquez, Stella M. Hartinger

**Affiliations:** 1 School of Public Health and Administration, Universidad Peruana Cayetano Heredia, Lima, Peru; 2 Tulane University School of Public Health and Tropical Medicine, New Orleans, Louisiana, United States of America; 3 Johns Hopkins Bloomberg School of Public Health, Baltimore, United States of America; 4 School of Science and Philosophy, Universidad Peruana Cayetano Heredia, Lima, Peru; 5 Swiss Tropical and Public Health Institute, Basel, Switzerland; 6 University of Basel, Basel, Switzerland; The University of Sydney, AUSTRALIA

## Abstract

Land use change (LUC) is a main cause of global environmental change, and is an important activity to be studied. Our research aims to examine the current state of evidence on the link between LUC and human health in the Amazon region. We conducted a scoping review of literature in two research databases, resulting in 14 papers for analysis. Our analysis demonstrated a lack of clear definitions for LUC, a wide variety of negative health effects from LUC, the lack of qualitative articles, a lack of studies exploring the potential positive health effects of LUC, and the predominance of studies coming from the Brazilian Amazon. Our study validated the prevailing idea that LUC can lead to negative health consequences, if not managed properly.

## Introduction

Considered one of the main causes of global environmental change, land use change (LUC) is generally defined as human modifications of land and the ways it is used (e.g. clearing forests for agriculture, infrastructure development, timber harvesting, among others) [1,2]. Changes in land use have multiple drivers, ranging from population growth, migration, and changes in governmental policies, to cultural changes influencing attitudes towards land, dietary transitions and incentives for forests conservation [3,4]. Usually, the goal of LUC is to obtain natural resources to fulfill human needs, which can result in negative impacts on the environment [5] and human health [6]. There is evidence that LUC has affected global water and carbon cycles, and global climate [5].

Worldwide, agricultural expansion, a component of LUC, remains the most substantial driver of deforestation rate: about 40% of deforestation in the tropics and subtropics is for large-scale commercial agriculture [7]. From 2010 to 2015, loss of forest area occurred mainly in the tropics, reaching 1770 million ha in 2015. During the same time period, Brazil ranged first in net tropical forest loss, accounting for 984,000 ha of net forest loss per year [3]. Deforestation in the Amazon, while not as prevalent as it used to be, increased by 29% between 2015 and 2016 [8]. 7,989 square kilometers of Brazil’s jungle were lost from August 2015-July 2016 [9,10], and between 2001 and 2015, 1,809,553 ha of forest have been lost in Peru [11].

Changes in ecosystems can have harmful consequences to human health. It is estimated that almost one quarter of the global burden of disease can be attributed to the environmental changes (including LUC) that contribute to air, water, and soil pollution [12,13]. Forest fragmentation and degradation due to LUC is threatening the Amazon Basin, one of the most biodiverse regions on earth and provider of key environmental services that contribute to global and local well-being. These impacts in turn affect human health through different pathways, for instance by changing vector population dynamics and pathogen transmission dynamics, potentially contributing to increased disease burden relating to vector borne diseases [13–16]. A variety of studies conducted in the Amazon region have described the health effects of LUC, both directly (e.g. measuring incidence of specific diseases on human populations) [1,2] and indirectly (e.g. measuring changes in vector population) [17,18]. However, there is no comprehensive review of the current state of evidence for the whole region.

Scoping reviews aim to broadly examine the existing literature on a topic in order to review concepts, report on the type of studies available regardless of their methodological quality, and identify gaps in knowledge to further inform research practice [19]. As populations continue to move into previously uninhabited areas, and as more research continues to reveal potential health effects of human activities, having comprehensive reviews on LUC and health topics will become vital to planners, policymakers, and those who work in the health industry. The aim of this scoping review is to identify articles that focus on LUC practices and their potential health outcomes within the Amazon region and identify gaps in knowledge to further inform research practice and policy makers.

## Methods

The steps included for our review followed the framework outlined by Arksey and O’Malley [20], taking into account recommendations made by Levac [21]: (1) identification of the relevant research question, (2) identification of relevant articles, (3) article selection, (4) charting of the data, and (5) collecting, summarizing and reporting of the results. The optional sixth step proposed in the framework, conducting a consultation exercise, was not carried out.

### Identifying the research questions

This review answered the following question: What is the current state of evidence on the link between LUC and human health in the Amazon region?

### Identification of relevant articles

We conducted a comprehensive scientific literature search in the electronic databases PubMed (biomedical sciences) and Web of Science (multidisciplinary). We developed and mapped key search terms with online databases prior to the article search. The research query included terms related to the Amazonian region, different aspects and activities related to LUC, and various types of diseases (Table 1). We carried out the search on September 6, 2016, where all articles were uploaded to a Zotero database [22].

**Table 1:** Keywords (with synonyms) and syntax used for literature search.

#1: "Amazon" terms	#2: "location" terms	#3: "land use" terms	#4: "health" terms	#5: combined search
(Amazon* OR "amazon basin") AND	(peru OR bolivia OR brazil OR columbia OR colombia OR venezuela OR "french guiana" OR "guyana" OR "surinam*" OR ecuador) AND	("land use" OR "land use change" OR deforestation OR mining OR mine OR agricultur* OR farm* OR road OR dam) AND	(health OR disease OR Dengue OR "yellow fever" OR "leishmaniasis" OR malaria OR arbovirus OR arenavirus OR leptospirosis OR hantavirus OR diarrhea* OR injur* OR accident* OR "mental health" OR neurological OR *nutrition OR anemia OR parasit* OR "parasite infection" OR "parasitic worm" OR *virus)	#1 AND #2 AND #3 AND #4

**Fig 1:**
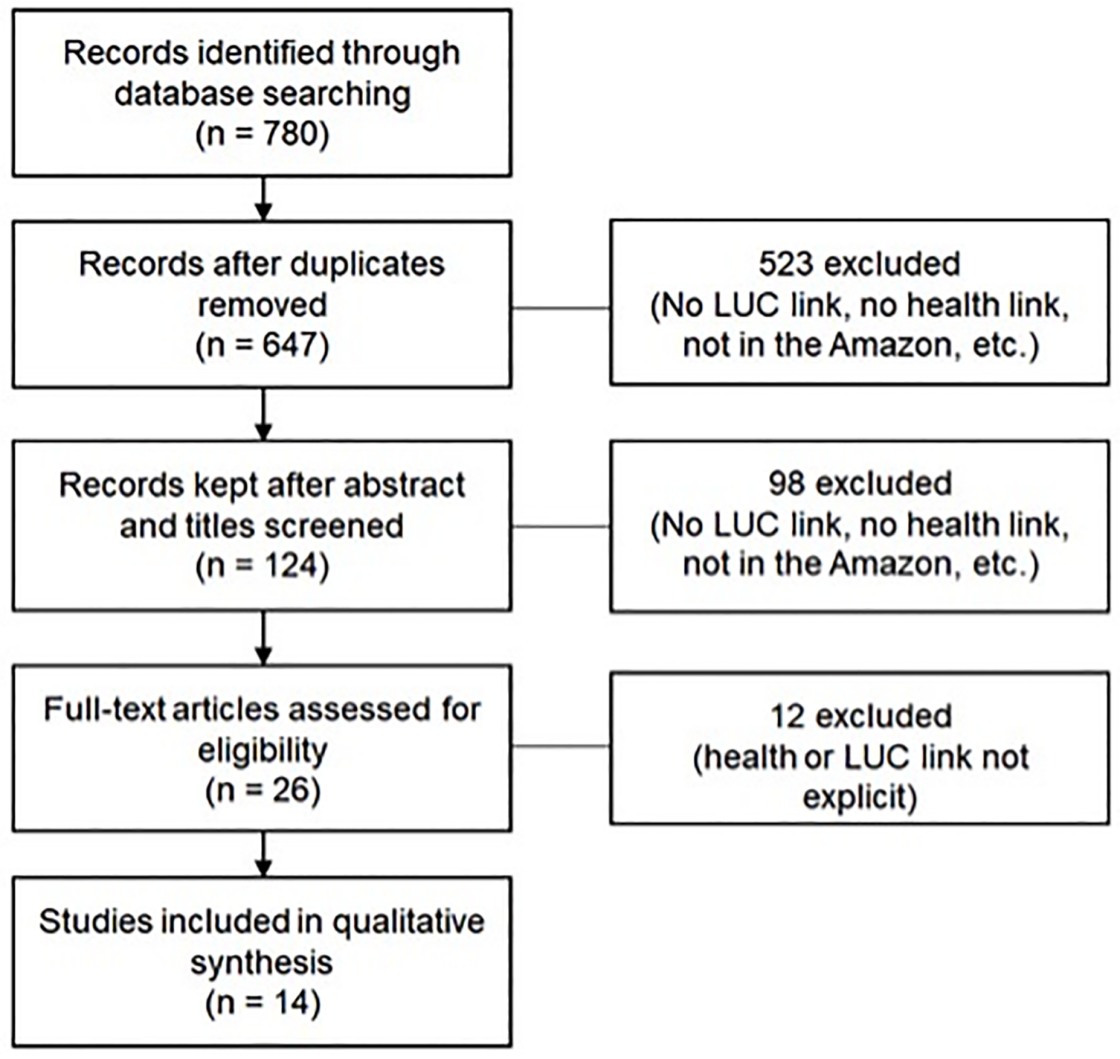
Flowchart. Flowchart of study selection process.

Our analysis for inclusion was a multi-step process (Fig 1). Our initial search yielded 780 papers, and after evaluating for the search criteria’s (LUC link, health link, etc.) we analyzed 14 articles. On March 16, 2017, from articles in our Zotero database, we created an initial list that met eligibility criteria by evaluating their titles and abstracts. At this step, we recognized that there was already a wealth of information on malaria and mercury (64 papers, including other reviews); therefore, we opted to exclude them from our paper. Selected articles were then read in full and evaluated for inclusion. Two reviewers independently conducted all stages of the scoping review, from relevance screening to data extraction. The two selectors individually selected the papers for each phase of elimination. Before moving on to the next stage, the selectors met and discussed each paper they chose to eliminate or keep until selectors reached agreement. Any discrepancy was discussed in a meeting with all co-authors to debate whether the article met our selection criteria or not.

### Article selection

We defined relevant publications as: any peer-reviewed article (except for reviews) published between January 1 2000 and August 31 2016; in English or Spanish; focused on or presenting data from the Amazon region (Brazil, Peru, Colombia, Venezuela, Ecuador, Bolivia, Guyana, Suriname, and French Guiana); that refer to a specific health issue; and include the topic of LUC. Our inclusion criteria for the topic of LUC was defined as either papers that refer to the existence of one or more aspects of LUC, or that partly or fully fulfilled the concept of land use change (LUC) as defined by the Intergovernmental Panel on Climate Change (IPCC): “…human activities which: (a) Change the way land is used (e.g., clearing of forests for agricultural use, including open burning of cleared biomass), or (b) Affect the amount of biomass in existing biomass stocks (e.g., forests, village trees, woody savannas, etc.)"[1].

We used papers that studied causative agents for health problems (e.g. vectors, arsenic exposure, air and water quality, etc.) for analysis, even if no health measurements were taken on humans. While it is not always true that the presence/absence of these factors will result in negative health outcomes, we felt this was a safe assumption based on existing literature [23]. Finally, we excluded articles on LUC that focused on topics that may be distally related to health outcomes, such as immigration (distally related to LUC, however after settlement).

### Data management and characterization/charting

Authors created an Excel spreadsheet [24] in which data extracted from the selected articles, including authors, year of publication, title, research objectives, location, study design, type of LUC and measures, and health outcome were recorded. In an additional column, authors recorded the summary of the findings from the articles.

### Analyzing, summarizing, and reporting the results

The analysis and synthesis of literature included quantitative analysis (e.g. descriptive statistics) and qualitative analysis (i.e. content analysis). A narrative approach allowed reviewers to extract common themes that emerged from the findings.

## Results

### Literature profile

Of the 26 articles reviewed in full, we included 14 in the analysis after exclusion criteria were applied. These articles pertain only to two Amazonian countries, Brazil and Peru, with the former providing the most articles (11). Health data referred to in these articles came from direct and indirect measurements of a disease in humans, vectors, or animals. Only one paper addressed disease as a risk through a measurement of air pollutant exposure. Three articles were based on epidemiological data (e.g. incidence, prevalence, mortality), 5 of them on data collected from vectors (e.g. relative abundance and richness of species), and one addressed local people’s perceptions of their wellbeing. Most papers did not describe specific methods for measuring changes in land use or forest cover. Five papers used mapping and satellite images to quantify forest cover; one used data on fire outbreaks related to deforestation, and one was based on governmental data on the average amount of square kilometers deforested per year. Over three quarters of reviewed articles were published from 2010 and on, reaching a peak with four publications in 2016. All were written in English.

**Fig 2:**
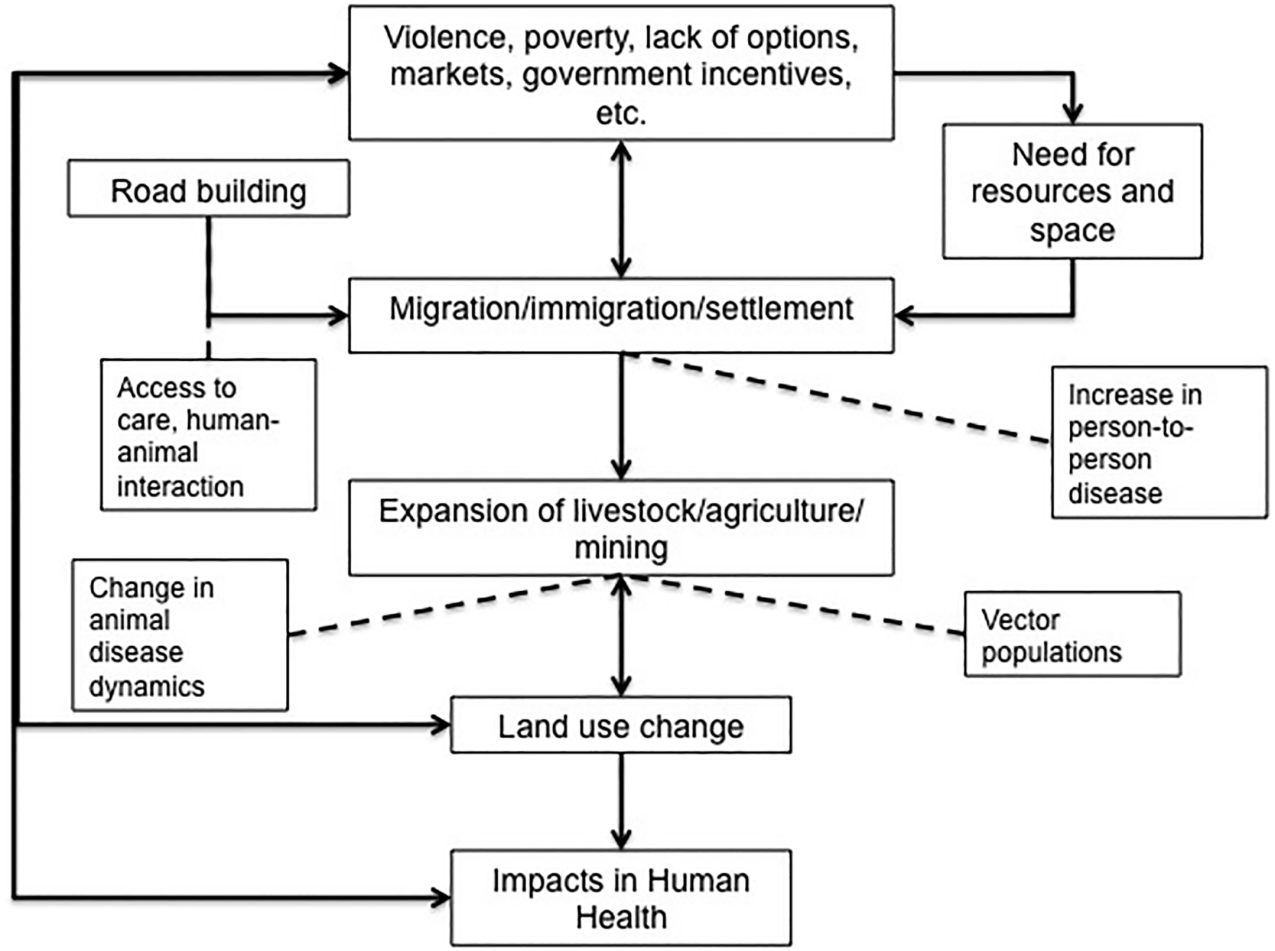
Factors relating to land use change. Conceptualization process for determining LUC and health topics.

The data collection table we used is shown in Table 2, and includes our results. Study characteristics are summarized in Table 3. Fig 2 depicts a conceptualization we developed to portray how we defined what we considered to be direct and indirect LUC and health topics; it describes how various specific LUC activities, human activities, and health effects are related. Table 4 outlines the methods used in each of the papers selected for review.

**Table 2:** Summary of selected publications.

Citation	LUC issue	Health issue	Location	concept of land use change	Results (summary)
Fernandez 2014 [28]	power plant construction	Schistosomiasis and other mollusk-related parasites	Brazil	none	Geographical distribution of known species expanded and recorded related to the construction of the dam. Reported species that could potentially spread Centerocestus formansus, schistosomiasis, and Brevifurcate apharyngeate cercaria.
Andrade 2016 [25]	Highway construction	rabies (bovine/human)	Brazil	contains the phrase "land use change" but doesn’t define or conceptualize it.	Areas of highest risk for bovine rabies found in proximity of rivers and highways. Highest concentration of high-risk for human rabies found where the highway network coincides with high densities of rural and indigenous populations. The high-risk areas for human and bovine rabies are patchily distributed, and related to extensive deforested areas, large herds of cattle, and the presence of highways.
Bauch 2015 [12]	mining, roads, and protected areas	malaria, diarrhea, and acute respiratory infection (ARI)	Brazil	Provides multiple references and mentions specific examples, but does not define or conceptualize it.	roads associated with increases in malaria and decreases in diarrhea and ARI cases. Only strict protected areas (PA) reduce incidence of all three diseases. Estimates suggest that expansion of strict PAs will reduce ARI, Diarrhea, and malaria.
Medeiros 2010 [26]	highway construction	Hantaviruses	Brazil	none	Areas studied were in influence area of BR-163 highway in Brazil. Results suggest the occurrence of non severe and/or atypical hantaviral infection cases, as well as the possible under-notification of cases. Recent circulation of hantavirus in one region, continuous exposure in three. Prevalence of anti-hantavirus antibodies similar to other states in some regions. Some seasonal and regional variations in prevalence. Evidence of multiple strains in some states.
Nascimento 2012 [33]	Biomass burning	Pneumonia (in children)	Brazil	none	Rates of admission due to pneumonia were distributed randomly across the municipalities, but fires were not. Fires mostly occurred in known areas of deforestation, suggesting anthropogenic biomass burning. There were not more admissions in the arc of deforestation.
Ramos 2014 [27]	agriculture	Leishmaniasis	Brazil	none	Abundance seemed to be related to human population presence and forest cover. Greatest abundance of sand flies and greatest richness of species found in areas with highest human population density. Highest index diversity in environments with high population and high forest cover, and environments with low population density and low forest cover. Abundance of 4 species of medical importance detected.
Reinhardt 2001 [28]	Biomass burning	Respiratory problems	Brazil	none	Residents in the rural village under study were exposed to significant smoke during the dry season burning period. The concentrations of particulate matter were above air quality standards for PM10 and PM2.5. CO levels were comparable to those of a moderately polluted urban setting and benzene and HCHO levels elevated when compared to rural areas in other parts of the world.
Roque 2013 [34]	agriculture	Chagas	Brazil	none	Higher prevalence of *T*. *cruzi* found in areas of low and intermediate disturbance, and higher prevalence in domestic mammals found in intermediate disturbance areas. Exposure of dogs to T. cruzi infection was the common feature among studied localities, stressing importance of using domestic mammals as important identifiers of T. cruzi hotspots.
Salmon-Mulanovich 2016 [29]	highway construction	community perceptions health and rodent borne diseases	Peru	land use change keyword, and the phrase is used through the paper, examples given	Survey information reported that 90% of participants recognized that rodents transmit diseases, but most couldn’t name a disease. Rodents were described as a pest rather than a reservoir or vector. Participants perceived an increase in the amount of rodents after the construction of the highway and in coming migration to the area.
Silva 2009 [35]	biomass burning	Asthma	Brazil	none	Spatial distribution of hospitalizations for asthma resembles the configuration of the “arc of deforestation”, however the incidence rate of hospitalizations is decreasing. Peak of hospitalizations in March and May in all states.
Streicker 2016 [36]	Agriculture	Rabies	Peru	Phrase is mentioned in title and through paper, examples given.	Bat bites common where there was no livestock, suggesting a protective effect of livestock on humans. Bats have more diversity in pretty where there is intermediate environmental disturbance with low densities of livestock where wildlife are more abundant. Feeding patterns demonstrated may increase transmission of rabies from bats to wildlife, which could create new rabies reservoirs or intermediate hosts for human infections.
Yanoviak 2006 [30]	agriculture	Mosquito borne diseases	Peru	Phrase is mentioned	Deforestation (and the resulting land use change after) increase the availability of micro-habitats for mosquitoes via increasing amounts of fallen plant parts. The fallen plants and water-filled plant axils can increase habitats favored by mosquitoes that may transmit Bunyamwera-group viruses and related pathogens. Preliminary identification suggests presence of species that includes main vector of Oropouche virus.
Reddington 2015 [32]	Biomass burning	PM emissions (air quality) / Premature adult mortality from cardiopulmonary disease and lung cancer	Brazil	none	Reductions in Brazil’s deforestation rates have caused reduced fire emissions resulting in improved air quality with positive impacts on human health. Reduction in particulate matter may be preventing roughly 400 to 1,700 premature adult deaths annually across South America.
Guimaraes 2016 [31]	highway construction	prenatal and childbirth assistance access	Brazil	none	The indicators of prenatal care and child delivery were below the national average, showing that geographical isolation still affects women’s health care in the Amazon, despite the construction of the highway and governmental health protocols adopted during this period.

**Table 3:** Basic details of included publications.

Paper characteristics	Number (n = 14); Included papers, n (%)	Article number in references
Type		
Quantitative research	13 (92.9)	[28], [25], [12], [26], [33], [27], [28], [34], [35], [36], [30], [32], [31]
Qualitative research	1 (7.1)	[29]
Country of origin		
Brazil	11 (78.6)	[28], [25], [12], [26], [33], [27], [28], [34], [35], [32], [31]
Peru	3 (21.4)	[29], [36], [30]
Publication year		
2000–2005	1 (7.1)	[28]
2006–2010	3 (21.4)	[26], [35], [30]
2011–2016	10 (71.5)	[28], [25], [12], [33], [27], [34], [29], [36], [32], [31]
Health topic		
Schistosomiasis etc.	1	[28]
Rabies	2	[25], [36]
Multiple	1	[12]
Hantavirus	1	[26]
Lung health	4	[33], [28], [35], [32]
Leishmaniasis	1	[27]
Chagas	1	[34]
Community perceptions on disease	1	[29]
Mosquito diseases	1	[30]
Healthcare Access	1	[31]
Land use change topic		
Agriculture	4	[27], [34], [36], [30]
Power plant construction	1	[28]
Multiple topics	1	[12]
Highway construction	4	[25], [26], [29], [31]
Biomass burning	4	[33], [28], [35], [32]

**Table 4:** Data collection sources/instruments used in selected papers.

type of data collection	number of papers	citations
Animal collection	5	[28], [27], [29], [34], [36], [30]
Questionnaires/observations/surveys/etc	6	[26], [27], [29], [36], [30], [31]
Existing data on human death, hospitalization, or illness	6	[25]*, [12]*, [33]*, [35]*, [32]*, [31]*
Mapping/satellite/etc.	5	[25]*, [12]*, [33]*, [27]*, [32]*, [30]
Air quality sampling	1	[32]

Items in Table 4 with no asterisk are primary data. Items with an asterisk are secondary data.

### The link between land use change and health

Eight out of 14 papers described negative relationships between LUC and health [25–32], one depicted positive [33], and five demonstrated neutral/mix correlations [13,34–37]. The most common health issue was lung health [13,29,33,34,36], followed by mosquito-borne illnesses [13,31] and rabies [26,37].

#### Road building

Road building is considered a particularly damaging form of LUC in tropical forests [38] and was the most commonly addressed in the analyzed articles [13,26,27,30,32]. Andrade et al. (2016) found that the presence of highways was related to a higher risk of contracting bovine and human rabies in the state of Pará, Brazil [26]. The presence of the Brazilian BR-163 highway (Cuiabá-Santarém Highway) was associated with an increased prevalence of specific antibodies against hantaviruses, increased incidence of hantavirus pulmonary syndrome, and emergence of new hantavirus lineages [27]. Authors ascribed this to an increased probability of contact between humans and rodents (vectors of hantaviruses) due to activities related to the highway pavement and construction itself, and deforestation fostered by economic activities (e.g. agriculture, farming, wood exploitation) developed along the influence area of the highway. Similarly, Salmon-Mulanovich et al., (2016) reported a perceived increase in rodent population after the paving of the Interoceanic Highway (IOH) along eight communities in the state of Madre de Dios, Peru, where participants mentioned seeing rodents in their *chacras* (small agricultural plot of land), communities, and houses. By easing access to healthcare services, roads were associated with a decrease of diarrhea and acute respiratory infections (ARI) in Brazil [13]. The same paper also found an increase in malaria cases due to roads, likely due to ecosystem disturbances that led to more human-mosquito interactions [13].

There have been mixed positive and negative results when examining highway construction and child/maternal health. Despite the construction of the Pacific Highway in Assis, Brazil, women still had low numbers of prenatal appointments. While the women in the community had access to more appointments than the rest of the state, the access was lower overall compared to the rest of Brazil. The addition of a road allowed for easier access to cesarean procedures, but there was no increase in them, suggesting that they were only used when medically necessary. There also continued to be a low number of institutionalized births (compared to the national average), suggesting the continued use of in-house childbirth. [32].

#### Biomass burning

Multiple papers looked at lung diseases [29,33,34,36]. Brazil has implemented burning and deforestation restrictions in many areas in order to minimize poor air quality impacts and to help control escaped fires. While these laws can result in improved air quality and reduction of premature adult deaths [33], other studies have found that people are still potentially exposed to significant pollution in affected areas. In Theobroma, Rondônia, measurements taken during burning season found particulate matter (PM_10_ and PM_2.5_) levels above Brazilian and/or U.S National Ambient Air Quality standards, CO levels comparable to urban areas (despite sampling taking place in a rural village), and elevated benzene and HCHO levels when compared to other rural areas around the world [29]. Another paper reported hospitalizations for children for asthma in the Brazilian Amazon increased in the arc of deforestation, though this could be explained by the rainy season and the resulting increase in fungi and mites [36]. In contrast, when assessing the number of admissions due to pneumonia and its relationship with fires in the Mato Grosso, one study [34] found that hospital admissions were randomly distributed through the municipalities, showing no apparent correlation with biomass burning [34].

#### Dam power plants

Only one paper discussed LUC related to dam power plants—these were associated with abundance of potential vector species of snail [25]. These snails had the potential to spread *Centerocestus formasus*, schistosomiasis, and the parasite responsible for cercarial dermatitis [25].

#### Livestock farming

When assessing papers with LUC due to livestock farming and health outcomes, one paper reported that bat bites on humans were more common where there was no livestock, suggesting that livestock could have a protective effect on bat bites on humans [37]. Bats fed on both humans and wildlife in areas of the Amazon affected by deforestation, which shows potential dietary switching [37]. The results from these studies give rise to concerns of increased bat-to-wildlife transmission of rabies in the amazon, which may potentially create more intermediate hosts or reservoirs for the disease and maintenance of wild rabies in the region. Similarly, the Marajó region, an area with a low and widely dispersed population, had a large number of human rabies cases in 2004 that coincided with a reduction in cattle herds, most likely causing an increase on human feeding by bats as they sought new food sources. There is more bat-borne rabies in humans in the Brazilian Amazon than there is in the rest of Latin America, which could be explained by the dispersed populations and proximity to bat food sources. The overall high-risk areas for both human and bovine rabies are deforested areas, areas with livestock, and highways [26].

#### Agriculture

LUC related to agriculture was associated with multiple diseases. A study of different Brazilian localities found that the prevalence of *Trypanosoma cruzi* infection was higher in the Genipaúba locality (characterized by low human occupation and less environmental degradation than Ajuaí), and the Ajuaí locality (characterized by low human occupation, and some degradation through fruit harvest and plantations), as compared to other districts that have high human occupation, nearby secondary vegetation, and areas of only farm and pasture. There was also higher prevalence of detected antibodies among mammals and domestic mammals in Ajuaí compared to other study sites [35].

*Phlebotomine* sandflies, potential carriers for leishmaniasis, were found in equal abundance across areas with different degrees of human occupations and land degradation. All sand fly species in this study were found in similar numbers through the land use categories suggesting high adaptability of sandflies, and the high adaptability could increase human-vector interactions, leading to more disease [28]. Deforestation, and the resulting land use changes, were found to increase the presence of potential mosquito habitats, either through more fallen plant parts or through plant axils that could serve as refuge [31].

#### Forest conservation

The changing of land from unprotected forest to protected areas was associated with a decrease in malaria, diarrhea, and ARI [13]. There are many possible explanations for this: the halt of deforestation decreasing interactions between people and forested areas that contain or can spread the diseases; a decrease in smoke emission from fires for deforestation; air filtration by trees; more clean water for hygiene; and filtration and water purification through forested areas by natural processes [13].

## Discussion

The scoping review utilized a systematic approach to explore associations—both positive and negative—between health problems and LUC in the Amazon forest of Latin America. The most striking observations were the lack of clear definitions for LUC, the lack of qualitative articles, a lack of studies exploring the potential positive health effects of LUC, and the predominance of studies coming from the Brazilian Amazon. Slightly more than half of the articles examined in this study revealed that LUC can lead to negative health consequences (beyond those excluded from this analysis that are associated to malaria and mercury), but other articles in our review also focused on the fact that there are often positive consequences associated with LUC. Gaps in knowledge include lack of agreed definition for LUC, a lack of representation for many different health outcomes, and the need for more research beyond the Brazilian Amazon.

### The concept of land use change

LUC is becoming a growing interdisciplinary field of study, receiving increasing attention from scholars within both environmental and social sciences. Academic and non-academic organizations are designating LUC as a core concern, thus potentially fostering research to better understand its causes and results.

There are multitudes of ways to examine LUC, from physical observations of cover change to studies on the ways humans interact with their ever-changing world [39]. The present review focused on LUC within the tropical rainforest, and indeed there is a great deal of research on these topics in places such as Southeast Asia and Central America. However, many other types and categories of LUC exist, and having clear definitions of land use and land use cover classes is important in order to collect data more efficiently and to use and build modeling tools [40]. With such a breadth of focus in this field, the use of “land use change” as a keyword, and defining it, would make seeking information within these varied focuses easier. It could aid in helping investigators to explain complex, interrelated factors that may affect their research.

Another important task for the research community would be to work on the development of a framework—or various frameworks—that portray the complex linkages associated with LUC—whether some are causal relationships or co-emerging issues. For example, one of our difficulties was determining what articles to include in this review, since the linkages between LUC and health are sometimes direct, and at other times more indirect. Having a framework that is used by the various researchers working on this topic would allow us to contribute to building the knowledge about these linkages, modifying the frameworks based on findings if appropriate, and exploring complex topics regarding attribution and causality. Another reason to have a clear framework is that it could aid in helping direct us on issues to examine—information that would serve vulnerable populations who often have the most at stake in LUC activities. Rural populations, especially indigenous ones, are at a higher risk for exploitation by the government and corporations when it comes to LUC activities, through both the co-opting of land and through the unsafe and unhealthy conditions that can often result from LUC [1]. These underserved populations have the most to gain from LUC and health research. Underserved and rural populations can also be major drivers of land use change themselves as they expand into new territories for property, food, and other resources. It is vital that stakeholders and organizations work with the local communities to also help them understand the cost-benefits of LUC so they can make informed decisions about their land.

Though most of reviewed papers address changes in land use by reference to deforestation, none of them defined, and therefore measured, LUC explicitly. Streiker & Jacob (2016) and Bauch et al. (2015) present the concept in their titles and/or abstracts, and approach LUC beyond deforestation, providing multiple examples and/or references to both causes and consequences of LUC (such as urbanization, agricultural intensification, climate change and ecosystem change). Other articles, [26,30,31], while listing LUC as keyword, do not provide an explicit definition. Thus, we found most of the papers (10) via the specific LUC topic keywords, instead of by the term “land use change” in and of itself.

### Health outcomes

Health effects related to smoke exposure and air quality were the most frequently addressed topic in the reviewed articles, especially in Brazil. This could possibly be explained by the multitude of laws regarding biomass burning that are now in effect in Brazil [33], and the desire to see their effectiveness. In contrast, there were no studies addressing food security and nutrition, mental health, and accidents or injuries, demonstrating a lack of representation of these issues among studies that link LUC to wellbeing and health.

Many of the papers we examined studied human-vector interactions. This is unsurprising, as vectors are a major cause for disease and can be relatively easily measured. There are also many examples of diseases transmitting as a result of increased contact between human population and wildlife, such as Ebola [41] and yellow fever [42].

Only one qualitative research satisfied our inclusion criteria [30]. This should not be interpreted as an absence of qualitative research—although very few studies using qualitative methods came across in the initial search—but rather that they are not well represented when researching the link between LUC and health. The drivers of LUC are human-based, and usually revolve around people seeking better opportunities through livelihoods. Some studies [30,43] show that while people living in newly settled areas know that land degradation is bad, they believe that the economic opportunities outweigh the negatives. Knowing this can help organizations shape their outreach programs and policy recommendations to ensure that people feel economically stable while still securing a healthy environment.

### Importance of more geographical variety in studies

The Amazon Basin encompasses 8 different countries (Brazil, Bolivia, Peru, Ecuador, Colombia, Venezuela, Guyana, Suriname and French Guiana) [44]; however, nearly all the papers we reviewed were about studies in Brazil. The majority of the Amazon rainforest is in Brazil, so its importance in management cannot be ignored. However, the differences between countries and areas of the Amazon in land use, human demographics, economic use, environment, and regulations call for more geographical diversity in Amazonian studies.

The differences in ecosystems across the Amazon basin make it difficult to apply one study to the entire region. While it is tempting to characterize the Amazon as one giant rainforest, it is made up of multiple ecosystem types, including mangroves, moist forest, mountain forest, and swamps [45]. This diversity in ecosystem leads to dramatic biodiversity, which can lead to different diseases spread to humans via the vast differences in plant and animal species.

When working with any health topic, the diversity of people is important to consider. The indigenous people of the Amazon are made up of about 350 ethnic groups, some of which are still isolated [46]. Amazonian tribes that are geographically close can sometimes have very different diets, as the foods harvested and consumed are a means of cultural expression. This can lead to not only nutritional differences, but differences in disease exposure [47].

### Drivers of land use change

The drivers of LUC, and subsequent relationships with the government, also can vary. The illegal logging and mining industries of the Amazon are a looming presence. For example, in Colombia, almost all forest clearing is illegal. However, Colombia has made strides in its protection of indigenous lands, giving ownership of the majority of the remaining Amazon to its indigenous peoples [48]. Brazil, having the higher GDP and more of the Amazon, has a higher potential to impact the rainforest than other states. Part of this is apparent in the construction of the trans-oceanic highway, which had significant foreign influence from important economic partners [49]. In Suriname, indigenous communities are working to create protected areas that make up almost half of Suriname, and contains much of the regions forest [50].

### Limitations

One of our barriers was language. There were 24 papers that were written in Portuguese. Because we had no Portuguese-speakers on our team, we were unable to include these papers, limiting our results. Spanish publications were underrepresented. The addition of grey literature would have enriched our findings; however, due to limited human resources and time constraints, we were unable to search these. As described previously, another problem the researchers encountered when conducting this search was drawing specific conclusions whether an article covered a specific LUC issue, and to what extent the LUC occurred, making drawing conclusions difficult.

## Conclusion

LUC will continue to be an important research topic, both globally and locally, as human expansion continues and shifts. These changes have the potential to do great harm to humans, but with continued research and advocacy, those harms could be mitigated. Our research found that most papers lack a clear definition of LUC, demonstrate mostly negative impacts on human health, and few papers study qualitative aspects of LUC. Moving forward, the authors recommend offering clear definitions of LUC and the way it is measured, exploring the social dimensions of LUC, and performing more qualitative studies to assess these better.

## Supporting information

S1 Table Complete list of articles from all search stages. (XLSX)

S1 File PRISMA checklist. (DOC)
